# An Association Study Between Educational Attainment-related Genes and Cognitive Functions in Japanese Patients with Schizophrenia Based on Full Pleiotropy

**DOI:** 10.14789/jmj.JMJ23-0009-OA

**Published:** 2024-02-10

**Authors:** NARIHIRO ORIMO, NARIMASA KATSUTA, WANYI MAO, ERIKO FUKUSHIMA, KAORI KAWAHARA, KEN NAKAYAMA, HITOKI HIROSE, HIROKI YAMASHITA, SHOHEI NISHIMON

**Affiliations:** 1 Department of Psychiatry, Juntendo University Graduate School of Medicine, Tokyo, Japan; 1 Department of Psychiatry, Juntendo University Graduate School of Medicine, Tokyo, Japan; 2 Health & Safety Promotion Center, Juntendo University, Tokyo, Japan; 2 Health & Safety Promotion Center, Juntendo University, Tokyo, Japan

**Keywords:** schizophrenia, pleiotropy, educational achievement, JART, WAIS-R

## Abstract

**Objectives:**

This study presents the multifaceted effects of candidate loci identified by genome-wide association studies on parameters such as educational background and the clinical symptoms of Japanese patients with schizophrenia along with detailed psychological measurements. This study aimed to investigate whether gene mutations that affect cognitive dysfunction are (1) related to the onset of schizophrenia and (2) also affect cognitive dysfunction in patients with schizophrenia.

**Design:**

Case-control study.

**Methods:**

This study evaluated 12 single-nucleotide polymorphisms (SNPs) (rs10189857, rs2175263, rs9398171, rs12670234, rs6466056, rs11156875, rs2018916, rs11663602, rs11885093, rs9404453, rs2473938, and rs4275659) that are common in Japanese individuals and demonstrated a relationship with schizophrenia and educational attainment in a previous genome-wide study. We included 640 Japanese patients (schizophrenia group) and 640 healthy participants (control group). Both groups were investigated for the relationship between the SNPs and educational attainment as well as psychometric evaluations of cognitive function.

**Results:**

The 12 SNPs were not identified as genetic risk factors for schizophrenia. However, rs9404453 was associated with a decline in educational achievement, educational performance, Japanese Adult Reading Test (JART100) score, and Wechsler Adult Intelligence Scale-Revised (WAIS-R) (full-scale intelligence quotient [FSIQ]) score in patients with schizophrenia, SNP rs6466056 was associated with a decline in the WAIS-R (FSIQ) score, and SNP rs11663602 was associated with a decline in the JART100 score.

**Conclusion:**

The SNPs rs9404453, rs6466056, and rs11663602 may be associated with academic performance or cognitive decline in patients with schizophrenia, although the overall findings from psychological tests did not show the expected consistency.

## Introduction

Schizophrenia (SCZ) is a typical psychiatric disorder with a prevalence of approximately 1%. SCZ exhibits several genetic features, which often overlap for long periods of time because the disorder presents with positive symptoms, such as hallucinations and delusions, as well as negative symptoms, such as autism, withdrawal, and social dysfunction^1, 2)^. The etiology and pathophysiology of SCZ may include multiple genetic and environmental factors; however, the underlying mechanisms remain unclear. The fact that the incidence of SCZ in identical twins is 50% and that in children born to the same parents is tenfold higher strongly suggests the involvement of heredity.

The advancements in genetic analysis technology since 2000 have allowed genome-wide association studies (GWASs) based on gene polymorphisms, and recent GWASs have suggested that certain candidate gene regions are associated with SCZ. Moreover, some neuropsychological tests, including the Wechsler Adult Intelligence Scale-Revised (WAIS-R)^3, 4)^, the Japanese Adult Reading Test (JART; Japanese version of the National Adult Intelligence Test for Estimating Premorbid Intelligence)^5, 6)^, and the Frontal Lobe Cognitive Function Test^7-9)^, have identified intellectual and cognitive dysfunction in patients with SCZ.

Several reports have evaluated the relationship between SCZ and educational achievement^10, 11)^, and a GWAS of correlation with educational achievement has been performed on a large sample^12)^. Leveraging GWASs to identify genetic correlations between complex traits and diseases can help elucidate the pathophysiology of diseases^13)^. In this regard, one study demonstrated that three independent single-nucleotide polymorphisms (SNPs; rs9320913, rs11584700, and rs4851266) are important throughout the genome^12)^. Similarly, in our previous study, the genetic region of 2q32.3 was suggested to affect educational achievement and cognitive decline in SCZ^14)^. Another GWAS identified 10 loci that were shared between SCZ and college level, and 29 loci that were shared between SCZ and years of education^15)^, while a GWAS meta-analysis revealed new loci and genetic correlations for general cognitive function, providing new insights into the genetics of neurocognitive function^16)^.

While previous studies have demonstrated that selected loci are associated with cognitive dysfunction and SCZ, we hoped to determine whether similar results could be obtained in Japanese patients with SCZ. Therefore, this study investigated whether such gene mutations that affect cognitive dysfunction are (1) related to the onset of SCZ and (2) also affect cognitive dysfunction in SCZ.

## Methods

### Participants

This case-control genetic association study included 640 unrelated Japanese patients with SCZ (302 men, 328 women; mean age ± SD = 38.1 ± 11.4 years). The diagnosis of SCZ was confirmed according to the criteria of the Diagnostic and Statistical Manual of Mental Disorders-V (DSM-V) after a structured clinical interview with the patient. Patients with schizoaffective disorders or mood disorders were excluded. In addition, 640 healthy individuals (322 men, 318 women; age, mean ±SD = 43.3± 11.9 years) were recruited from Saitama and Tokyo and included in the study as a control group. Healthy individuals were defined as those who did not meet the current or past criteria for any Axis I disorder (DSM-V). Overall, patients in both groups met the following criteria: (1) absence of systemic or neurological diseases, (2) absence of head trauma complicated by loss of consciousness, and (3) no medical history of dependency on alcohol or other substances. The mean age was significantly lower in the SCZ group than in the control group (p < 0.001), whereas the sex distribution was comparable between the two groups (p = 0.14). Notably, the number of subsamples that underwent assessments for both SCZ and cognitive functioning was 252 in both cases.

### SNP selection and genotyping

Peripheral white blood cells were used to extract genomic DNA with help of the QIAamp^®^ DNA Blood Maxi Kit (Qiagen, Courtaboeuf, France). Twelve SNPs (rs10189857, rs2175263, rs9398171, rs12670234, rs6466056, rs11156875, rs2018916, rs11663602, rs11885093, rs9404453, rs2473938, and rs4275659) that are common in Japanese individuals and had demonstrated relationships with SCZ and educational attainment in a previous GWAS^15)^ were investigated in the present study. We limited our analysis to the 12 loci whose expression frequency in Japanese individuals was estimated to be more than 20% as per the Thermo Fisher website (https://www.thermofisher.com/jp/ja/home/brands/thermo-scientific.html). We excluded loci with low expression frequencies in the Japanese population because we considered the number of samples in our cohort to be extremely small. The SNP analyses were performed using TaqMan^®^ technology (Assay-by-Design™) on an ABI7500 system (Applied Biosystems, Foster City, CA, USA). However, the probes and primers were developed by the Assay-by-Design™ service (Applied Biosystems). Polymerase chain reaction (PCR) was performed with a standard PCR MasterMix reagent kit in a 4-µL volume. The SNP analysis results were validated by using a direct DNA sequencing method (TaqMan^®^ method) in a few randomly selected participants to check for errors. The results from direct sequencing were similar to those obtained using the TaqMan^®^ method for all investigated SNPs. The general and common information (e.g., gene name and position) of the selected SNPs is provided in Supplementary table 1.

### Clinical and cognitive assessments

Experienced psychiatrists interviewed patients and their family members to evaluate their clinical symptoms. These interviews were conducted at the beginning of the study period and if the patients showed any new acute symptoms during the study period. Psychiatrists also examined the patients using a cognitive test battery. Age at disease onset was defined as the age at the first presentation of any SCZ symptom reported in the DSM-V, and was determined on the basis of interviews with the patients and their family members, in addition to the relevant medical records. Daily antipsychotic doses were converted to chlorpromazine (CP)- equivalent doses^17)^. Clinical symptoms were evaluated using the Brief Psychiatric Rating Scale (BPRS), in which each item was rated on a 7-point scale, and the total rating was compared between the groups^18)^.

Social adjustment and cognitive function assessments were performed after the patients’ severe symptoms improved from the time of admission. Comprehensive Assessment of Symptoms and History (CASH)^19)^ and the Modified Premorbid Adjustment Scale (MPAS)^20)^ were used to evaluate social status. For the “current occupation” and “previous occupation” items in CASH, we simply used the classifications of “employed” or “unemployed” because this item was difficult to analyze. Cognitive assessments were performed using WAIS-R^21)^ to assess present intelligence, JART^22)^ to evaluate premorbid intelligence, and verbal fluency tests^23, 24)^ and the Stroop test^25, 26)^ for assessment of the prefrontal cortex and cognitive functions^27)^.

### Statistical analysis

A two-tailed Student’s t-test was used to compare the mean values of continuous variables, while categorical variables were compared using Chi-square (χ^2^) test. All statistical analyses were performed using SPSS Statistics software version 21 (IBM, Chicago, IL, USA). For the case-control association study, Hardy-Weinberg equilibrium (HWE) tests for the SNPs were performed using SNPAlyze software version 7.0 Pro (Dynacom, Yokohama, Japan). The HWE tests were performed for all loci in both groups. All statistical significance values were two-tailed and were analyzed using Bonferroni correction (probability level of p < 0.05/3 SNPs = 0.0167 in each analysis). Power was calculated using a prevalence rate of <0.01 with an additive or multiplicative model, assuming various degrees of allelic frequencies and odds ratios for the SNPs.

The Kruskal-Wallis test was performed to highlight potential differences in clinical characteristics (three genotyped patient groups for each SNP). Accordingly, a post-hoc analysis was conducted using the two-tailed Mann-Whitney U test. As a method to analyze the relationship between SNP and each parameter, we selected the multiple linear regression analysis, following previous our research^14)^. A correlation test was performed to detect the factors related to differences in clinical characteristics among the genotypes based on their correlations with altered clinical characteristics. As a result, only potentially significant factors (independent variables) were included in the multiple linear regression analysis for potentially significantly different clinical characteristics among the genotypes (dependent variables) using genotypes as dummy variables (0/1; e.g., G/G = 1, A/G, and A/A = 0).

## Results

### Genetic case-control analyses

Twelve SNPs were genotyped in both the study groups, and the completeness of genotyping was between 99.0% and 99.6%. No deviations from the HWE were observed in either group (all p > 0.05; Table 1). None of the SNPs showed significant associations between their allelic or genotypic frequencies and SCZ.

**Table 1:** Distribution and statistical analysis of educational attainment SNPs in Japanese patients with schizophrenia

Genotype frequency (%)				p-value	HWE c/s	Allele frequency (%)		χ^2^	p-value	Odds ratio (95%CI)
rs2175263	A/A	A/T	T/T	0.6882	0.942/0.421	A	T	0.3433	0.5579	1.0439 (0.904-1.205)
Schizophrenia	188 (26.3)	362 (48.5)	196 (26.3)			738 (49.5)	754 (50.5)			
Controls	174 (26.5)	374 (50.1)	198 (26.5)			722 (48.4)	770 (51.6)			
rs2473938	C/C	C/G	G/G	0.0958	0.005/0.197	C	G	3.4864	0.0619	0.8639 (0.741-1.007)
Schizophrenia	62 (8.21)	336 (44.5)	357 (47.3)			460 (30.5)	1050 (69.5)			
Controls	67 (9.01)	366 (49.3)	310 (41.7)			500 (33.6)	986 (66.4)			
rs4275659	C/C	C/T	T/T	0.0221	0.069/0.325	C	T	6.9734	8.27E−03	0.8113 (0.695-0.948)
Schizophrenia	379 (50.5)	317 (42.2)	55 (7.32)			1075 (71.6)	427 (28.4)			
Controls	328 (43.6)	355 (47.1)	70 (9.30)			1011 (67.1)	495 (32.9)			
rs6466056	C/C	C/T	T/T	0.8285	0.060/0.281	C	T	0.0541	0.8161	1.0199 (0.8639-1.2041)
Schizophrenia	420 (55.6)	292 (38.8)	40 (5.32)			1132 (75.3)	372 (24.7)			
Controls	421 (55.9)	297 (39.4)	35 (4.65)			1139 (75.6)	367 (24.4)			
rs9398171	C/C	C/T	T/T	0.2037	0.4288/0.257	C	T	1.1008	0.2941	0.9156 (0.7765-1.0796)
Schizophrenia	45 (6.00)	303 (40.4)	402 (53.6)			393 (26.2)	1107 (73.8)			
Controls	49 (6.57)	268 (35.9)	429 (57.5)			366 (24.5)	1126 (75.5)			
rs9404453	A/A	A/G	G/G	0.9192	1/0.807	A	G	0.1213	0.7276	1.0273 (0.8827-1.1956)
Schizophrenia	313 (42.6)	336 (45.7)	86 (11.7)			962 (65.4)	508 (34.6)			
Controls	309 (42.0)	335 (45.6)	91 (12.4)			953 (64.8)	517 (35.2)			
rs11156875	A/A	A/G	G/G	0.4601	0.398/0.343	A	G	1.5964	0.2064	0.9089 (0.7837-1.0541)
Schizophrenia	301 (40.7)	332 (44.9)	107 (14.5)			934 (63.1)	546 (36.9)			
Controls	282 (37.8)	344 (46.1)	120 (16.1)			908 (60.9)	584 (39.1)			
rs11663602	A/A	A/C	C/C	0.5352	0.108/0.770	A	C	0.4309	0.5115	0.9531 (0.8256-1.1002)
Schizophrenia	172 (22.3)	369 (49.3)	207 (27.7)			713 (47.7)	783 (52.3)			
Controls	189 (25.3)	351 (47.1)	206 (27.6)			729 (48.9)	763 (51.1)			
rs11885093	C/C	C/T	T/T	0.0646	0.011/0.564	C	T	0.3119	0.5765	1.0443 (0.8969-1.2161)
Schizophrenia	333 (44.3)	340 (45.3)	78 (10.4)			1006 (67.0)	496 (33.0)			
Controls	337 (45.7)	299 (40.6)	101 (13.7)			973 (66.0)	501 (34.0)			

HWE, Hardy-Weinberg equilibrium; CI, confidence interval; c/s, controls/schizophrenia

### Genotype effect on clinical characteristics

In the SCZ group, 252/640 patients were admitted to Juntendo Koshigaya (Saitama) or Juntendo Hospital (Tokyo) due to acute symptom exacerbation. The inpatients underwent both clinical and cognitive assessments. However, not all of these 252 patients could be examined using all cognitive assessment tests. For instance, although the BPRS scores could be estimated in all 252 patients, some patients could not be examined using complicated assessments such as the WAIS-R. Furthermore, some clinical information could not be easily evaluated on the basis of the information obtained from patients and their family members. For instance, the evaluation of parental educational achievements in CASH could not be easily performed for patients with deceased parents. Thus, the number of patients included in the analysis of each clinical variable was different, and the detailed case numbers for each test in the battery are shown in Table 2.

**Table 2:** Clinical characteristics and test scores in each genotype from study participants

Variables	Patients	rs6466056			rs9404453			rs11663602		
		C/C	C/T	T/T	A/A	A/G	G/G	A/A	A/C	C/C
	*n = 252	*n* = 150	*n* = 93	*n* = 9	*n* = 108	*n* = 109	*n* = 35	*n* = 71	*n* = 119	*n* = 62
**Clinical variables**		Mean ± SD (min-max)								
Sex, M/F		77/73	41/52	6/3	51/57	56/53	17/18	40/31	53/66	31/31
Age, mean ± SD, (years)		37.0 ± 12.9	36.5 ± 14.2	30.1 ± 7.6	38.1 ± 13.3	35.5 ± 13.2	35.3 ± 13.2	36.6 ± 11.2	36.3 ± 14.1	37.0 ± 13.9
		(15-76)	(14-76)	(19-43)	(14-76)	(16-76)	(17-63)	(17-63)	(14-76)	(15-76)
Onset (years)		25.2 ± 8.8**	23.1 ± 8.8**	20.0 ± 5.8**	24.7 ± 8.6	23.6 ± 8.6	24.9 ± 10.1	23.7 ± 7.2	24.2 ± 9.3	24.9 ± 9.5
		(14-76)	(13-53)	(13-30)	(13-52)	(11-53)	(12-54)	(13-53)	(13-54)	(11-53)
Post hoc analysis (Mann-Whitney U tests)		C/C vs T/T; χ^2^ = −1.748, *p* = 0.081								
		C/C vs C/T; χ^2^ = −2.201, *p* = 0.028								
		T/T vs C/T; χ^2^ = −0.826, *p* = 0.409								
Duration of disease (years)		12.6 ± 10.1	13.0 ± 10.3	9.6 ± 4.8	14.1 ± 10.2	11.9 ± 10.0	10.5 ± 9.3	14.0 ± 11.1	11.7 ± 9.3	12.9 ± 10.1
		(0-48)	(0-39)	(1-19)	(0-41)	(0-48)	(0-35)	(1-48)	(0-41)	(0-41)
DUP (months)		17.7 ± 22.6	14.0 ± 20.4	23.3 ± 33.0	16.8 ± 22.8	15.2 ± 21.2	19.9 ± 24.1	18.7 ± 24.1	14.7 ± 21.4	17.5 ± 21.9
		(0-96)	(0-96)	(0-96)	(0-96)	(0-96)	(0-96)	(0-96)	(0-96)	(0-96)
CED (mg/day)		1021.2 ± 544.1	1057.6 ± 641.9	766.1 ± 210.3	1070.7 ± 661.1	1015.3 ± 491.8	919.3 ± 533.2	986.3 ± 478.5	1027.9 ± 628.4	1066.2 ± 577.6
		(2-2760)	(150-4400)	(350-1065)	(120-4400)	(200-2300)	(2.7-1975)	(3-2050)	(200-4400)	(150-2460)
**Clinical symptoms**										
BPRS scores (total)	*n = 252	*n* = 150	*n* = 93	*n* = 9	*n* = 108	*n* = 109	*n* = 35	*n* = 71	*n* = 119	*n* = 62
		35.8 ± 11.3	36.1 ± 12.7	40.4 ± 10.4	35.0 ± 12.7	37.2 ± 10.4	35.7 ± 12.9	35.1 ± 11.3	35.8 ± 11.7	37.6 ± 12.4
		(0-82)	(0-84)	(31-63)	(0-63)	(10-84)	(20-82)	(0-82)	(0-84)	(0-63)
**Social status**										
CASH	*n = 190	*n* = 116	*n* = 66	*n* = 8	*n* = 79	*n* = 81	*n* = 30	*n* = 51	*n* = 90	*n* = 49
Current employed/unemployed		107/10	62/6	6/2	74/7	71/11	30/0	46/5	89/5	42/8
Previous employed/unemployed		46/67	29/36	2/5	34/45	33/43	12/18	18/30	41/52	20/26
Educational achievement of subject		12.4 ± 2.3	12.5 ± 2.5	11.6 ± 1.2	12.7 ± 2.3**	12.0 ± 2.4**	12.7 ± 2.3**	12.0 ± 2.3	12.4 ± 2.5	12.9 ± 2.3
		(9-21)	(8-20)	(9-13)	(8-20)	(9-18)	(9-21)	(9-20)	(8-21)	(9-18)
		Post hoc analysis (Mann-Whitney U-tests)			A/A vs G/G; χ^2^ = −0.507, *p* = 0.612					
					A/A vs A/G; χ^2^ = −2.444, *p* = 0.015					
					G/G vs A/G; χ^2^ = −1.574, *p* = 0.115					
Educational performance		3.1 ± 0.9	2.8 ± 0.9	3.8 ± 1.7	3.0 ± 0.8**	3.2 ± 1.0**	2.7 ± 1.0**	3.0 ± 1.0	3.0 ± 1.0	3.2 ± 0.8
		(1-6)	(1-5)	(2-7)	(1-5)	(1-7)	(1-6)	(1-5)	(1-7)	(1-5)
		Post hoc analysis (Mann-Whitney U-tests)			A/A vs G/G; χ^2^ = −1.851, *p* = 0.064					
					A/A vs A/G; χ^2^ = −1.627, *p* = 0.104					
					G/G vs A/G; χ^2^ = −2.879, *p* = 0.004					
Educational achievement of parents		11.6 ± 3.7	13.2 ± 3.2	13.0 ± 3.1	12.0 ± 3.8	12.1 ± 3.1	13.4 ± 3.7	12.1 ± 2.9	12.5 ± 3.7	12.0 ± 3.8
		(2-21)	(6-21)	(9-16)	(2-21)	(2-16)	(9-21)	(6-16)	(2-21)	(2-18)
		rs6466056			rs9404453			rs11663602		
		C/C	C/T	T/T	A/A	A/G	G/G	A/A	A/C	C/C
**Psychometrics**										
**Frontal lobe function**	*n = 113	*n* = 62	*n* = 45	*n* = 6	*n* = 46	*n* = 49	*n* = 18	*n* = 30	*n* = 55	*n* = 28
Verbal fluency test		24.8 ± 11.6	24.7 ± 9.8	24.9 ± 10.1	26.1 ± 12.0	22.9 ± 9.5	27.1 ± 11.2	23.3 ± 12.9	24.9 ± 10.1	26.1 ± 9.9
		(5-67)	(7-49)	(9-39)	(6-67)	(5-49)	(9-45)	(6-67)	(5-54)	(8-50)
Stroop test (time)		105.4 ± 51.6	102.5 ± 60.6	126.5 ± 75.2	108.1 ± 51.8	105.3 ± 66.5	98.5 ± 35.4	94.6 ± 48.1	101.9 ± 51.9	123.7 ± 69.3
		(10-271)	(−6-335)	(59-231)	(15-220)	(−6-335)	(37-208)	(−6-195)	(10-271)	(24-335)
**Intelligence scales**	*n = 64	*n* = 34	*n* = 25	*n* = 5	*n* = 20	*n* = 31	*n* = 13	*n* = 19	*n* = 31	*n* = 14
JART 100		87.1 ± 19.1	89.2 ± 18.4	92.6 ± 11.4	89.6 ± 18.0**	84.1 ± 17.8**	97.3 ± 18.5**	81.1 ± 16.6**	92.1 ± 18.0**	88.4 ± 19.3**
		(55-121)	(57-122)	(81-107)	(57-114)	(55-118)	(68-122)	(57-114)	(55-121)	(55-122)
		Post hoc analysis (Mann-Whitney U-tests)			A/A vs G/G; χ^2^ = −1.482, *p* = 0.138			A/A vs C/C; χ^2^ = −1.601, *p* = 0.109		
					A/A vs A/G; χ^2^ = −1.526, *p* = 0.127			A/A vs A/C; χ^2^ = −2.81, *p* = 0.005		
					G/G vs A/G; χ^2^ = −2.656, *p* = 0.008			C/C vs A/C; χ^2^ = 0.36, *p* = 0.717		
WAIS-R (FSIQ)		71.3 ± 13.6**	79.2 ± 13.3**	72.7 ± 23.8**	79.4 ± 12.7**	69.8 ± 13.7**	78.4 ± 17.9**	76.1 ± 14.2	76.3 ± 16.0	68.6 ± 12.3
		(48-114)	(58-110)	(44-110)	(60-110)	(44-110)	(52-114)	(52-110)	(48-114)	(44-89)
Post hoc analysis (Mann-Whitney U tests)		C/C vs T/T; χ^2^ = −0.133, *p* = 0.894			A/A vs G/G; χ^2^ = −0.221, *p* = 0.825					
		C/C vs C/T; χ^2^ = −2.533, *p* = 0.011			A/A vs A/G; χ^2^ = −2.720, *p* = 0.007					
		T/T vs C/T; χ^2^ = −0.826, *p* = 0.409			G/G vs A/G; χ^2^ = −1.654, *p* = 0.098					
(VIQ)		77.6 ± 13.7	83.9 ± 17.4	78.2 ± 20.0	84.5 ± 16.6	75.8 ± 13.0	83.7 ± 19.4	81.5 ± 14.9	81.9 ± 17.1	74.6 ± 14.1
		(53-119)	(48-121)	(55-113)	(53-121)	(55-113)	(48-119)	(53-119)	(55-121)	(48-97)
(PIQ)		70.1 ± 14.6	74.2 ± 16.1	78.8 ± 20.1	77.0 ± 11.8	69.5 ± 14.6	72.0 ± 21.6	75.0 ± 14.3	73.9 ± 15.2	65.4 ± 17.2
		(49-103)	(29-97)	(54-103)	(61-97)	(49-103)	(29-103)	(54-97)	(49-103)	(29-97)

BPRS, Brief Psychiatric Rating Scale; CED, chlorpromazine-equivalent dose; DUP, duration of untreated psychosis; JART, Japanese version of the National Adult Reading Test; FSIQ, full score intelligence quotient *Because some patients were difficult to be examined by complicated assessments, such as WAIS-R, and some correct clinical information was difficult to obtain from patients and their family (e.g.,., Educational Achievement of Parents of CASH because of the death of parents). Thus, the numbers of patients in each clinical variable were different. **p-values with statistical significance among the genotypes are presented in bold; then, post hoc analysis was performed between two genotype combinations.

### Social status

Among the eight subscales of CASH, only “Educational Achievement of Subject” and “Educational Performance” scores showed statistically significant differences correlated with the rs9404453 genotype, with p-values of 0.035 and 0.01, respectively. The A/A genotype demonstrated significantly higher “Educational Achievement of Subject” scores than the A/G genotype (χ^2^ = −2.444, p = 0.015) (Table 2). Moreover, the A/G genotype also showed a significantly higher “Educational Performance” score than the G/G genotype (χ^2^ = −2.879, p = 0.004). However, none of the subscale scores differed significantly in relation to the rs6466056 and rs11663602 genotypes. The MPAS, total BPRS, and Frontal Lobe Cognitive Function test scores were comparable among the genotypes of each of the three SNPs (Table 2).

### Psychometrics

The SNP rs6466056 showed a significant difference in relation to the full-scale intelligence quotient (FSIQ) values (χ^2^ = 6.109, p = 0.047), and post-hoc tests with Bonferroni correction showed that the FSIQ values of individuals with the C/C genotype were significantly lower than those of individuals with the C/T genotype (χ^2^ = −2.533, p = 0.011). The SNP rs9404453 showed a significant difference in relation to the JART score (χ^2^ = 7.548, p = 0.023) and FSIQ values (χ^2^ = 7.826, p = 0.020). Post-hoc tests showed that the JART score and FSIQ values of individuals with the A/G genotype were significantly lower than those of individuals with the G/G genotype, with p-values of 0.008 and 0.007, respectively. The SNP rs11663602 showed a significant difference in relation to the JART score (χ^2^ = 7.897, p = 0.019), and post-hoc tests showed that the JART score of individuals with the A/A genotype was significantly lower than that of individuals with the A/C genotype (χ^2^ = −2.81, p = 0.005). Performance IQ (PIQ) and verbal IQ (VIQ) values were comparable among the three genotypes (Table 2).

### Multiple linear regression analysis

To identify possible confounders affecting cognitive function, multiple regression analyses were performed with the BPRS score, educational history, and CP-equivalent dose at discharge for each SNP. The multiple regression analysis was performed on the basis of a previous study^14)^. Specifically, for rs6466056, the independent variables were age, BPRS score, education history, CP-equivalent dose, and each SNP, and the dependent variable was the total IQ value. For rs9404453, the independent variables were age, BPRS score, education history, CP-equivalent dose, and each SNP, and the dependent variable was the total IQ value. For rs11663602, the independent variables were age, BPRS score, education history, CP-equivalent dose, and each SNP, and the dependent variable was the JART100 estimated IQ value.

rs6466056 showed a significance probability < 0.05 for the dependent variable total IQ value. The significance probability was highly relevant, being 0.000 for the BPRS score and 0.013 for rs6466056. The standardized coefficients were −0.428 for the BPRS score and 0.287 for rs6466056, with the former having a slightly higher impact. The multiple regression equation was as follows: total IQ = BPRS score × (−0.685) + rs6466056 × 8.689 + 95.445. The R value was 0.541, while the adjusted R^2^ value was 0.267, and the goodness-of-fit of the multiple regression equation was low. We hypothesized that these findings indicated an association. In the stepwise method, the CP-equivalent dose at hospital discharge did not vary in relation to the JART100 score or the total IQ value.

rs9404453 also showed a significance probability < 0.05 in relation to the dependent variable total IQ value. The significance probability was highly relevant, being 0.001 for the BPRS score and 0.011 for rs9404453. The standardized coefficients were −0.388 for BPRS and −0.302 for rs9404453, with the former having a slightly greater impact. The multiple regression equation was BPRS × (−0.621) − rs9404453 × 9.041 + 101.013. The R value was 0.545; the adjusted R2 value was 0.272, and the goodness- of-fit of the multiple regression equation was low.

No multicollinearity was observed between rs11663602 and educational history for the dependent variable JART100 estimated IQ, with an R value of 0.435. The significance probability was <0.05. Educational background and rs11663602 were highly relevant, with significance probabilities of 0.000 and 0.005, respectively. The standardized coefficients were 0.341 for educational background and −0.236 for rs11663602, with the former having a slightly higher impact. The multiple regression equation was as follows: JART100 estimated IQ = Education × 2.46 − 9.731 × rs11663602, which was +61.24. However, the goodness-of-fit of the multiple regression equation was low, with an R value of 0.435 and an R2 value of 0.176.

## Discussion

In this study, we examined 12 SNPs (rs10189857, rs2175263, rs9398171, rs12670234, rs6466056, rs11156875, rs2018916, rs11663602, rs11885093, rs9404453, rs2473938, and rs4275659) that had been previously associated with educational attainment in a GWAS. We also determined the education- related clinical characteristics of Japanese patients with SCZ.

Our results demonstrate that these SNPs were not risk factors for the development of SCZ in Japanese patients. This finding is consistent with most previous GWASs, since the gene region where these three SNPs are located has not been identified as a genetic risk factor for SCZ.

Three of the 12 SNPs investigated in this study- rs6466056, rs9404453, and rs11663602-showed some degree of difference in the clinical features of SCZ related to education or cognitive functioning. rs6466056 produced significant differences in WAIS-R scores; rs9404453 in years of education, academic achievement, and JART100 and WAIS-R scores; and rs11663602 in JART100 scores. Of these, rs9404453 does not encode a gene, and its clinical significance is unknown. However, rs6466056 and rs11663602 act as introns of *SRPK2* and *KCNG2*, respectively, although their clinical significance is unknown.

SRPK2 is a serine/arginine-rich splicing factor (SRSF, a protein encoded by the SRSF gene and an essential factor in pre-mRNA splicing) and functions as a protein kinase involved in neuronal apoptosis^28)^ and the pathogenesis of Alzheimer’s disease^29)^. The *KCNG2* gene encodes a subunit of the voltage-gated potassium channel Kv6.2, is involved in neurotransmitter release and neuronal excitability, and has been implicated in opioid dependence^30)^.

For rs6466056, a significant difference was observed in all IQ test results but no significant difference was observed in JART scores, suggesting an association between disease status and IQ decline. For rs9404453, a significant difference was observed in all IQ test and JART100 scores. rs11663602 was also associated with significant differences in JART100 scores, suggesting that, similar to rs9404453, it is associated with lower pre-disease IQ.

The results of the stepwise multiple regression analysis suggested that the presence of these SNPs was related to IQ and JART scores, independent of CP-equivalent doses and BPRS scores. The WAIS-R and JART100 scores represent the IQ after and before the onset of SCZ^31)^, respectively, suggesting that the presence or absence of these SNPs affects IQ. Since the CASH contains many categorical variables, its findings were categorized by employment status for convenience. We then examined each SNP, but found no statistically significant differences due to the small number of samples. Moreover, none of the patients with the G/G phenotype for rs9404453 were employed at present.

This study had several limitations. First, patients using drugs other than antipsychotics were excluded. Second, although the WAIS-R was used to measure IQ, the clinical features of SCZ include impairments in verbal comprehension, working memory, perceptual organization, and processing speed. However, these variables are also measured using the WAIS-IV and may be correlated with the PIQ. Currently, we are collecting data on these four variables using the WAIS-IV to investigate their relationships with SNPs in a future study. Finally, we selected 12 candidate SNPs from the largest recent GWAS to show the relationship between these SNPs and both SCZ and educational background. Additional investigations are needed to validate the role of SNPs in cognitive dysfunction in patients with SCZ. The association between SNPs and intelligence in healthy controls was not investigated in this study, and the influence of other factors on the association between SNPs and intelligence cannot be ruled out. In addition, the number of samples for each psychological test was different because all patients who underwent psychological tests were inpatients, and a considerable number of these patients could not complete the tests because of their complexity or the severity of their psychiatric symptoms. In addition, the Bonferroni correction was only applied within each SNP, and no correction for the multiplicity of the 12 SNPs overall was indicated. For the rs9404453 gene polymorphism, the A/A genotype was associated with a higher educational background than the A/G genotype, whereas the A/G genotype was associated with higher educational performance than the G/G genotype. However, both the JART100 and WAIS-R (FSIQ) results were poor for the A/G genotype. Although the results of multiple psychological tests were inconsistent, the Educational Performance values were not prioritized because of the possibility of chance results because of poor accuracy. This is because Educational Performance is a self-reported numerical value by patients and their families and is a relative evaluation score that does not represent absolute academic ability and cannot be said to have high validity.

In conclusion, 12 SNPs (rs10189857, rs2175263, rs9398171, rs12670234, rs6466056, rs11156875, rs2018916, rs11663602, rs11885093, rs9404453, rs2473938, rs4275659) that affect cognitive dysfunction as identified by GWASs were not related to the onset of SCZ. However, three of these SNPs may be associated with a decline in educational performance and cognitive function in patients with SCZ. Although some items showed significant differences in relation to some SNPs, the expected consistency was not obtained when evaluating the psychological tests as a whole.

## Ethics approval statement

All participants provided written informed consent before participation. This study was conducted in compliance with the Declaration of Helsinki of the World Medical Association and was approved by the Research Ethics Committee of Juntendo University (2015014).

## Data availability statement

The research data are not shared. The raw data used in the present study cannot be made publicly available because disclosure of personal data was not included in the research protocol of the present study. The data are not publicly available due to privacy and ethical restrictions.

## Funding

This work was supported by the Juntendo Institute of Mental Health (grant number: 201701).

## Author contributions

All the authors contributed to the conceptualization, design, and writing of this manuscript. All the authors have read and approved the final version of the manuscript.

## Conflicts of interest statement

The authors declare that there are no conflicts of interest.
